# Phase I trial of convection-enhanced delivery of nimustine hydrochloride (ACNU) for brainstem recurrent glioma

**DOI:** 10.1093/noajnl/vdaa033

**Published:** 2020-03-26

**Authors:** Ryuta Saito, Masayuki Kanamori, Yukihiko Sonoda, Yoji Yamashita, Kenichi Nagamatsu, Takaki Murata, Shunji Mugikura, Toshihiro Kumabe, Eva Wembacher-Schröder, Rowena Thomson, Teiji Tominaga

**Affiliations:** 1 Department of Neurosurgery, Tohoku University Graduate School of Medicine, Sendai, Japan; 2 Department of Diagnostic Radiology, Tohoku University Graduate School of Medicine, Sendai, Japan; 3 Brainlab AG, Munich, Germany

**Keywords:** brainstem, convection-enhanced delivery, nimustine hydrochloride, recurrent glioma, temozolomide

## Abstract

**Background:**

Treatment options for patients suffering brainstem gliomas are quite limited as surgery is not an option against intrinsic tumors at brainstem and chemotherapy generally failed to demonstrate its efficacy. Intracerebral convection-enhanced delivery (CED) is a novel approach for administering chemotherapy to patients with brain tumors. We present the results of phase I trial of CED of nimustine hydrochloride (ACNU), designed to determine the maximum tolerable concentration of ACNU, for patients with recurrent brainstem gliomas.

**Methods:**

Sixteen patients, aged 3–81 years old, suffering from recurrent brainstem gliomas, including diffuse intrinsic pontine glioma patients as well as patients with recurrent gliomas that originated from non‐brainstem sites, were enrolled in this trial between February 2011 and April 2016. The dose/concentration escalation trial included 3 dose/concentration groups (0.25, 0.5, and 0.75 mg/mL, all at 7 mL) to determine the safety and tolerability of CED of ACNU. Real-time monitoring of drug distribution was performed by mixing gadolinium-tetraazacyclododecanetetraacetic acid (Gd-DOTA) in the infusion solution. CED of ACNU was given in combination with oral or intravenous temozolomide chemotherapy.

**Results:**

CED of ACNU demonstrated antitumor activity, as assessed by radiographic changes and prolonged overall survival. The recommended dosage was 0.75 mg/mL. Drug-associated toxicity was minimal.

**Conclusions:**

Intracerebral CED of ACNU under real-time monitoring of drug distribution, in combination with systemic temozolomide, was well tolerated among patients with recurrent brainstem gliomas. The safety and efficacy observed suggest the clinical benefits of this strategy against this devastating disease. Based on this phase I study, further clinical development of ACNU is warranted.

**Key Points**

- Results of phase I trial of CED of ACNU for patients with recurrent brainstem gliomas are presented.
- CED of ACNU under real-time monitoring of drug distribution was feasible and well tolerated.

**Importance of the Study**

Intracerebral convection-enhanced delivery (CED) is an approach for administering chemotherapy to patients with brain tumors. We present the results of phase I trial of CED of nimustine hydrochloride (ACNU) for patients with brainstem recurrent gliomas. Sixteen patients suffering from brainstem recurrent gliomas were enrolled in this trial. The dose/concentration escalation trial included 3 dose/concentration groups (0.25, 0.5, and 0.75 mg/mL, all at 7 mL) to determine the safety and tolerability of CED of ACNU. Real-time monitoring of drug distribution was performed by mixing gadolinium-tetraazacyclododecanetetraacetic acid (Gd-DOTA) in the infusion solution. CED of ACNU was given in combination with oral or intravenous temozolomide chemotherapy. CED of ACNU demonstrated antitumor activity. The recommended dosage was 0.75 mg/mL. Intracerebral CED of ACNU under real-time monitoring of drug distribution, in combination with systemic temozolomide, was well tolerated among patients with brainstem recurrent gliomas.

Brainstem gliomas account for 15–20% of childhood central nervous system tumors,^1^ and up to 85% of these cases are diffuse intrinsic pontine gliomas (DIPGs).^2^ Radiation is the standard treatment for DIPG, though it is the most therapeutically challenging of the brainstem gliomas subtypes. Despite collaborative efforts to improve treatment, the survival rate for DIPG has remained static over the last 20 years at about 10 months (median survival), with a 2-year survival rate below 10%.^3^ Although the prognosis is somewhat better than that for pediatric DIPG, recurrent adult DIPG and recurrent malignant glioma affecting the brainstem are similarly challenging.^4^

Local delivery is a promising treatment strategy for DIPG, as it bypasses the blood-brain barrier (BBB) enabling delivery of the highest drug concentration possible. However, given that drug distribution after local delivery depends on the diffusion properties of the infusates, successful distribution is challenging.^5^ Convection-enhanced delivery (CED) was developed in the early 1990s by Bobo et al.^6^ as a method that allows large and small molecular weight compounds to bypass the BBB. CED enables the robust distribution of molecules at the site of infusion and holds promise as an effective treatment strategy for brain tumors. In this study, we aimed to develop an effective therapeutic strategy against brainstem recurrent glioma with local CED.

We have been developing a CED-based strategy to treat malignant gliomas using the water-soluble nitrosourea compound nimustine hydrochloride (ACNU). ACNU is a nitrosourea compound broadly used in Japan against high-grade gliomas as a counterpart to BCNU before the era of temozolomide (TMZ). We first demonstrated the efficacy of ACNU delivered via CED using rodent intracranial xenografted tumor models.^7^ Subsequently, we reported the efficacy of combining CED of ACNU with systemic TMZ.^8^ Most recently, we demonstrated the feasibility and safety of CED of ACNU with real-time magnetic resonance (MR) monitoring using non-human primates.^9^ In the present report, we demonstrate the safety and feasibility of CED of ACNU in patients suffering from recurrent gliomas affecting the brainstem, including DIPG. We also reported, in the previous report that the concentration, rather than dose, of the infusate limits local toxicity in this drug delivery method.^10^ Therefore, this study was designed to determine the maximum tolerable concentration using a fixed infusion volume of 7 mL.

## Materials and Methods

### Patients

Patients diagnosed clinically or radiologically with recurrent glioma in the brainstem were eligible for this study. Cases of recurrent diffuse brainstem glioma or gliomas originating from surrounding structure (eg, thalamus, cerebellum) and infiltrating brainstem were included. Histological diagnosis of the initial tumor was required for recurrent cases of glioma originating from surrounding structures but was not required for cases of brainstem glioma. Recurrent cases after treatment by the standard regimen, namely, radiation with or without TMZ, were eligible. Other inclusion criteria included no chemotherapy or radiotherapy in the previous 4 weeks and normal hematologic, hepatic, and renal functions. The exclusion criteria were serious pulmonary, cardiac, or other systemic disease; acute infection; the need for anticoagulation and/or antiplatelet therapy; or any other cancer, unless the patient had been disease-free for more than 5 years.

The study protocols were approved by the institutional review board at Tohoku University Hospital and were registered (UMIN000005125). After a full explanation of the protocol, written informed consent was obtained from all patients before enrollment. In cases where it was difficult to obtain the patient’s signature due to neurological deficits or age, written informed consent was obtained from a representative person after confirming the patient’s approval.

### Study Design

This study had a prospective phase I open-label, nonrandomized, dose/concentration-escalation design. Patients received 7 mL infusion of ACNU at one concentration. Three new patients were initially considered at each dose level, and each patient received one treatment. The starting dose was 0.25 mg/mL, which is 1/4 the concentration that led to complete tumor regression without toxicity in a rat glioma model. Subsequent dose levels were 0.5 and 0.75 mg/mL. If 3 patients at one dose level developed dose-limiting toxicity, the prior dose level was considered the maximum tolerated dose. Here, the dose-limiting toxicity was defined as any symptomatic deterioration that persisted more than a month and was attributable to treatment. In addition to CED of ACNU, patients received systemic TMZ with the protocol for recurrent tumors, namely, 150 or 200 mg/m^2^/day for 5 days starting from day 1 of CED (Supplementary Figure S1).

### Study Procedure

**Figure 1.**
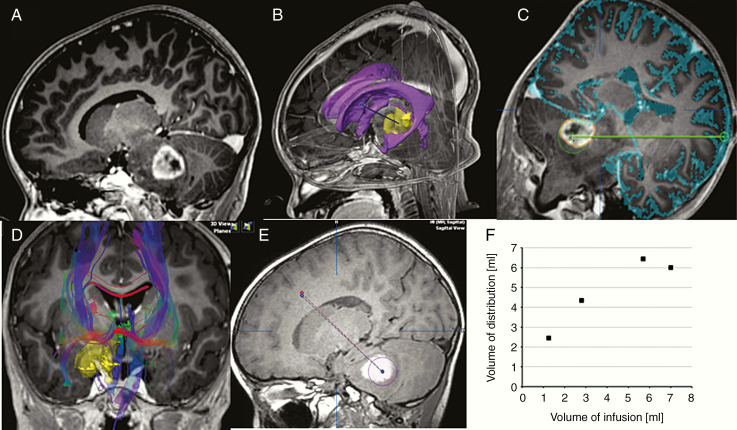
CED infusion in a representative patient. (A) Pre-infusion contrast-enhanced T1-weighted sagittal image. (B) Image showing the complex anatomy. Automatic object creation and fiber tracking can help define the clinical target volume, locate and avoid critical structures during surgical planning, and find the optimal trajectory. Yellow, tumor; purple, ventricle; blue-dotted line, planned catheter tract. (C) Automatic sulcus delineation overlaid on the anatomical images and geometrical visualization of the recommended distance from the catheter tip to avoid structures at risk and minimize the risk of leakage into CSF space. (D) Local detection of fiber bundles in the region of interest provides information about potential leakage pathways, thereby helping to identify an optimal trajectory. (E) Retrospective analysis of catheter placement accuracy. Planned trajectory (red) versus the actual catheter position (blue). Distribution of tracer agent (yellow outline). (F) The volume of distribution at each MRI time point plotted against the volume of infusion. *V*_d_/*V*_i_ was almost 2 at the beginning of infusion, but gradually slowed with *V*_d_ plateauing at 6–7 mL.

Our preoperative planning protocol consisted of an MR study with angiographs. In addition, we used a stereotactic computer tomography scanning dataset for localization. All datasets were imported and post-processed using iPlan Flow (Brainlab AG, Munich, Germany), and FDA-approved planning software specifically designed to support the planning of intracranial drug delivery catheters. Automatic co-registration of patient data enabled a multi-modality planning procedure. As a preparatory step, we fit the Leksell coordinate system to the plan and segmented the clinically relevant target volume, vessels, functional areas, cerebral spinal fluid (CSF) compartments, and sulci. To prevent loss of infusate to CSF space, we avoided placing the catheter close to the adjacent ventricles or the cortical surface. Specifically, a minimum distance of 10 mm from the catheter tip to CSF space was used as a guideline to minimize the risk of leakage in this study. iPlan Flow allowed geometrical visualization of the minimal catheter insertion depth in the form of a cylinder along the catheter track and the predefined minimal distance from the catheter tip to CSF space as a sphere around the catheter tip. The minimum insertion depth is dependent on the expected backflow length. Based on the infusion settings, the software estimates the expected backflow length and displays it as a cylinder along the trajectory. Within this cylinder length, we avoided a trajectory crossing any ependymal or pial surface. This information is displayed in 3 dimensions and provides instant feedback without the need for constant manual measurement. The impact of fiber tract orientation is critical for planning direct drug delivery in the brainstem, with previous work demonstrating that the infusate is preferentially distributed along perpendicularly oriented corticospinal tracts.^11^ iPlan Flow allows the import and analysis of diffusion tensor imaging. Automatically calculated color-coded maps provide information about the directionality of white matter tracts, which helped us to identify the potential leakage pathways while planning the trajectory (Figure 1). Up to 2 infusion catheters were inserted at coordinates optimized using iPlan Flow. Catheter placement was confirmed postoperatively by magnetic resonance imaging (MRI). The patients were then moved from the MRI environment and the infusion was administered in a monitored setting over 2.5 days. The infusion was regularly paused (4 times during the overall process) and patients were brought back into the MRI environment to monitor drug distribution. Monitoring infusion detects any leakage due to backflow along the catheter into undesired regions of the brain and allows for adjustment of the infusion protocol. Steroids and antibiotics were also administered throughout the infusion period.

### Safety Assessment

Before treatment, patients underwent baseline physical and neurological examinations and neuropsychological assessment. Vital signs and neurologic status were monitored throughout the infusions. Adverse events were classified in accordance with the National Cancer Institute Common Terminology Criteria for Adverse Events version 3.0. Patients were hospitalized for approximately 2 weeks and returned as outpatients for follow-up every 4 weeks. At follow-up visits, patients underwent physical and neurological examinations, including MRI with gadolinium-contrast enhancement to determine tumor response.

### Radiographic Assessment

Pretreatment MRI scans were obtained at Tohoku University Hospital an average of 4 days (range, 0–18 days) before ACNU infusion and at 4-week intervals after infusion. All scans were performed with T2-weighted spin-echo, T2-weighted fluid-attenuated inversion recovery, and T1-weighted multiplanar sequences before and after intravenous gadolinium infusion. Three-dimensional volumetric measurement of contrast-enhanced MRI was retrospectively conducted. Manual segmentation was performed using a region-of-interest analysis to measure tumor volume (in cm^3^) based on the contrast-enhancing tissue observed on the T1-weighted MRI. Responses were categorized according to the New Approaches to Brain Tumor   Therapy volumetric criteria reported in the study of Shah et al.^12^: complete response (CR, the disappearance of an enhanced lesion), partial response (PR, ≥50% decrease in the volume of contrast enhancement), progressive disease (PD, ≥25% increase in the volume of an enhanced lesion), and stable disease (SD, insufficient shrinkage to qualify for PR or insufficient increase to qualify for PD).

## Results

### Patient Characteristics

Sixteen patients with a median age of 34.5 (range 3–81) years were enrolled. Eleven patients had recurrent DIPG and 5 had recurrent high-grade glioma at the brainstem. Table 1 presents a detailed demographic, tumor, and prior treatment data. As we also recruited patients suffering from recurrent malignant glioma at the brainstem, patients with multiple recurrences and poor Karnofsky performance status score (KPS) were also included: 9 patients were at first recurrence and 7 were at more than the second recurrence, while 12 patients had KPS less than 70. The median and mean times from initial diagnosis to treatment with CED of ACNU were 7.73 and 16.6 months, respectively (range, 4–100 months).

**Table 1.** Patient Characteristics

Case	Age	Sex	Diagnosis	Primary Lesion	Previous Treatment	Recurrence Event/ Time From Diagnosis to Treatment	KPS at CED
1	37	F	GBM rec.	Multifocal (Rt Basal ggl, Rt T, Pons)	Removal + WB30GyLB30Gy + TMZ →GKR	2nd/5.1	50
2	49	M	GBM rec.	Rt Thal, Rt midbrain, Rt T	Biopsy + LB60Gy + TMZ → removal + TMZ	3rd/6.8	30
3	70	F	Pontine GBM rec.	Pons, Lt F	Biopsy + LB54Gy + TMZ → removal (frontal lobe)	2nd/6.8	40
4	46	M	GBM rec.	Rt T	Removal + LB40Gy + TMZ	1st/14	70
5	60	M	DIPG rec. (AA)	Pons	LB54Gy + TMZ	1st/9	60
6	51	M	AA rec.	Pineal	ACNU + WB30GyLB27Gy + TMZ + removal + removal + biopsy	3rd/28.6	50
7	81	M	GBM rec.	Lt P	Removal + LB60Gy + TMZ	1st/29.2	60
8	19	M	DIPG rec.	Pons	LB50.4 + TMZ + BEV	3rd/70.2	50
9	3	M	DIPG rec.	Pons	LB54Gy + TMZ	1st/5.7	60
10	7	M	DIPG rec.	Pons	LB54Gy + TMZ + VPshunt	1st/5.4	50
11	31	F	DIPG rec.	Pons	LB54Gy + TMZ	1st/33	90
12	40	M	DIPG rec.	Pons	LB50Gy + TMZ → BEV	2nd/28.6	60
13	32	F	DIPG rec.	Pons	LB54Gy	1st/4.2	80
14	3	F	DIPG rec.	Pons	LB54Gy + TMZ	1st/6.1	50
15	4	F	DIPG rec.	Pons	LB54Gy + TMZ → removal	2nd/3.5	50
16	6	F	DIPG rec.	Pons	LB54Gy + TMZ	1st/8.7	70

F, female; M, male; GBM, glioblastoma; DIPG, diffuse intrinsic pontine glioma; AA, anaplastic astrocytoma; rec, recurrence; Rt, right; Lt, left; Basal ggl, basal ganglia; T, temporal; Thal, thalamus; F, frontal; P, parietal; WB, whole-brain irradiation; LB, local brain irradiation; TMZ, temozolomide; GKR, gamma knife radiosurgery; BEV, bevacizumab; VPshunt, ventriculo-peritoneal shunt; KPS, Karnofsky performance status score.

### Feasibility of Real-Time MR Monitoring

In all 16 treatment cases, monitoring of infusion was possible by mixing 1 mM gadolinium-tetraazacyclododecanetetraacetic acid (Gd-DOTA). Data from a representative case are shown in Figure 1. Here, the analysis of serial MRI during infusion revealed that the distribution volume (*V*_d_) of the infusate first linearly increased and then plateaued with a volume of infusion (*V*_i_). Overall, the mean *V*_d_/*V*_i_ ratio was initially about 2 (Figure 1F) then gradually decreased with *V*_d_ reaching a steady state at 6–7 mL.

### Toxicities

In all 16 CED treatment cases, the full 7 mL volume was administered (Table 2). During CED of ACNU, 11 cases experienced worsening of their symptoms, likely due to edema caused by infusion of aqueous solution. With steroid use, 8 patients recovered within a week after stopping the infusion. In the first 3 patients (cases 1–3) who received 0.25 mg/mL ACNU (1.75 mg), no dose-limiting toxicity was observed. In the next 3 patients (cases 4–6) who received 0.5 mg/mL ACNU (3.5 mg), one patient had persistent deterioration of symptoms. This 46-year-old male patient suffered from a rapidly enlarging recurrent glioblastoma at the pons. Due to rapidly worsening left hemiparesis, he was barely able to walk before treatment; however, after treatment he was bedridden. As treatment failed to stop the enlargement of the tumor, his deteriorating condition may also be attributable to tumor growth; however, this was recorded as possible dose-limiting toxicity. Three additional cases (cases 7–9) were treated with 0.5 mg/mL ACNU without any persisting symptoms. One additional pediatric case (case 10) was also treated with 0.5 mg/mL ACNU to make sure the safety of this concentration in pediatric cases. Thereafter, the dose was escalated to 0.75 mg/mL (5.25 mg) in cases 11–16. At this level, possible dose-limiting toxicity was observed in 2 cases. One case was a 40-year-old male with recurrent DIPG. Similar to the previous case, he was suffering from rapidly deteriorating symptoms of hemiparesis and dysarthria before treatment. After treatment he could not walk and required assistance for daily activities. However, the speed of symptomatic deterioration decreased and he lived with maintained KPS for another 8 months after treatment. The second case was a 31-year-old female suffering recurrent DIPG with KPS 90 before treatment. During infusion, her motor weakness and dysarthria deteriorated and persisted until 2 months posttreatment.

**Table 2.** Results of Treatment

Case	Age	Sex	Diagnosis	Dose of ACNU (mg)	Deterioration of Symptoms During Infusion*	Consequences*	Best Response	OS After CED (M)	OS
1	37	F	GBM rec.	1.75	Somnolence G2, pyramidal tract dysfunction G3	Only transient	PD	5	10
2	49	M	GBM rec.	1.75	Somnolence G2, anorexia G2, pyramidal tract dysfunction G3, neuropathy CNX G3	Only transient	PD	6	13
3	70	F	Pontine GBM rec.	1.75	Neuropathy CNX G3, somnolence G2	Only transient	SD^1M^	5	12
4	46	M	GBM rec.	3.5	Somnolence G3, pyramidal tract dysfunction G3	Pyramidal tract dysfunction G3	SD^1M^	7	21
5	60	M	DIPG rec. (AA)	3.5	Ataxia G2, laryngeal nerve dysfunction G2	Only transient	SD^2M^	5	14
6	51	M	AA rec.	3.5	Seizure G2	Only transient	SD^3M^	16	44
7	81	M	GBM rec.	3.5	Pyramidal tract dysfunction G2	Only transient	PD	4	21
8	19	M	DIPG rec.	3.5	Fever G2, ataxia G3	Only transient	PD	2	71
9	3	M	DIPG rec.	3.5	(−)	None	PD	5	10
10	7	M	DIPG rec.	3.5	(−)	None	PD	4	10
11	31	F	DIPG rec.	5.25	Pyramidal tract dysfunction G2, neuropathy sensory G2	Pyramidal tract dysfunction G2, neuropathy CNX G2	SD^9M^	19	52
12	40	M	DIPG rec.	5.25	Neuropathy CNX G2, pyramidal tract dysfunction G3	Neuropathy CNX G2, pyramidal tract dysfunction G3	SD^5M^	8	36
13	32	F	DIPG rec.	5.25	Neuropathy sensory G2	Only transient	CR	29	33
14	3	F	DIPG rec.	5.25	(−)	None	PR	10	16
15	4	F	DIPG rec.	5.25	(−)	None	SD^3M^	6	10
16	6	F	DIPG rec.	5.25	(−)	None	PD	2	11

M, male; F, female; GBM, glioblastoma; DIPG, diffuse intrinsic pontine glioma; AA, anaplastic astrocytoma; rec, recurrence; CN, cranial nerve; G, grade; PD, progressive disease; SD, stable disease; PR, partial response; CR, complete response; OS, overall survival. The right upper case with “SD” indicates the duration (months) of SD diagnosis after CED.

*Common Terminology Criteria for Adverse Events v3.0.

### Response to Therapy

Two patients died only 2 months after the CED of ACNU. One was a 19-year-old male who experienced multiple recurrences and was treated with bevacizumab before enrollment in this trial (Supplementary Figure 2). Bevacizumab complicates the diagnosis of recurrence by attenuating enhancement. In this case, there was a strongly enhanced mass at the dorsal side of the pons as well as a slightly enhancing mass at the ventral side. As methionine positron emission tomography revealed high uptake at the slightly enhanced ventral side mass, the ventral side mass was targeted with CED of ACNU. However, the strongly enhanced mass rapidly enlarged and led to death. The other case was a 6-year-old female who developed urinary retention just 2 weeks after treatment. Spinal MRI was performed and revealed spinal dissemination (Supplementary Figure 3). We did not assess spinal MRI prior to treatment, but it is likely that the dissemination existed before CED.

**Figure 2.**
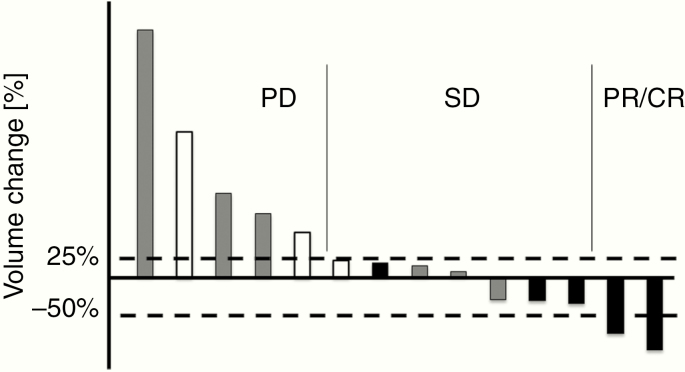
Waterfall plot of the maximum percentage change from baseline in the tumor volume of target lesions (*N* = 14). White boxes, patients received 0.25 mg/mL infusion; gray boxes, patients received 0.5 mg/mL infusion; black boxes, patients received 0.75 mg/mL infusion. Responses were categorized according to that of Response Assessment in Neuro-Oncology criteria.^12^

**Figure 3.**
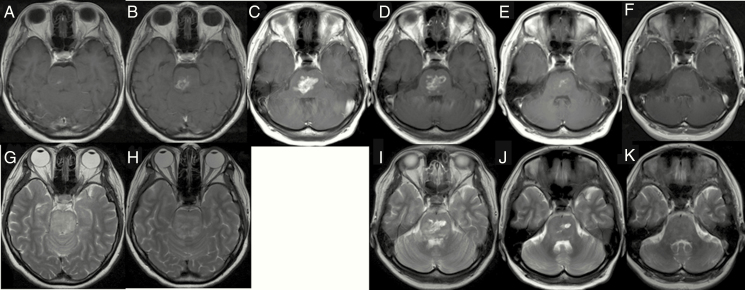
A case of a 32-year-old female treated for DIPG for 4 months prior to starting CED of ACNU. She was treated with local 54 Gy irradiation and then followed up at another hospital for her initial disease (A and G: 3 months before CED). She was referred to us 1 month prior to the CED of ACNU (B and H). Treatment was given against a rapidly enhancing mass (C). After treatment, TMZ monotherapy was continued (D and I) 2 months after CED (E and J: 5 months after CED). Her symptoms of truncal ataxia, diplopia, and dysarthria gradually recovered. Imaging revealed complete remission of the tumor at 7 months after the CED of ACNU (F and K). (A–F) T1-weighted images with contrast enhancement and (G–K) T2-weighted images.

**Figure 4.**
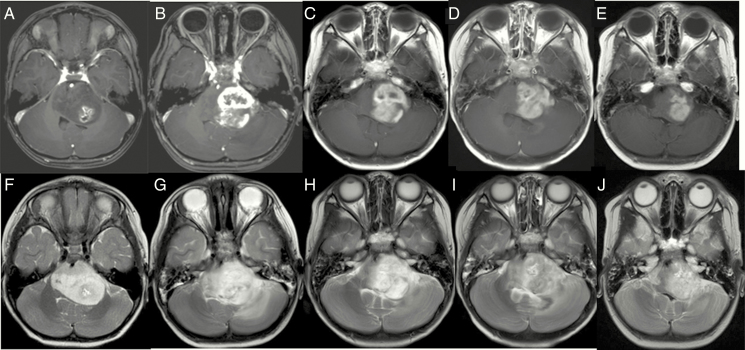
A case of a 3-year-old female treated for DIPG for 6 months prior to starting CED of ACNU (A and F). She was treated with local 54 Gy irradiation plus concomitant TMZ and followed up at another hospital. Due to growing contrast enhancement, she was referred to us and underwent CED of ACNU (B and G). After treatment, TMZ monotherapy was continued. Her symptoms of truncal ataxia, diplopia, and right hemiparesis gradually improved. Images reveal the responses until 3 months after CED of ACNU (C and H: 1 month after CED; D and I: 2 months after CED; E and J: 3 months after CED). (A–E) T1-weighted MRI with contrast enhancement and (F–J) T2-weighted images.

The best response to therapy for the remaining 14 cases is expressed in a waterfall plot in Figure 2. Results for a lower dose/concentration of infusion may be unsatisfactory. In the 3 cases that received 0.25 mg/mL ACNU (1.75 mg dose) infusion, treatment failed to control tumor enlargement. In the 7 cases that received 0.5 mg/mL ACNU (3.5 mg dose) infusion, 3 cases showed SD and the others showed PD. In the 6 cases that received 0.75 mg/mL ACNU (5.25 mg dose) infusion, 1 case showed PR,1 case showed CR, and 3 cases showed SD. The one adult DIPG case showing CR and one pediatric DIPG case showing PR are presented in Figures 3 and 4, respectively. The former case survived for 29 months after treatment while the latter survived for 10 months after treatment.

## Discussions

Gliomas infiltrating the brainstem are therapeutically challenging. Among the 16 cases that participated in this study, 6 cases were recurrent malignant glioma. Among these, 3 had multifocal diseases from the initial presentation. Although the tumor is multifocal, the brainstem lesions become the life-limiting disease in these cases. There were also 2 cases of recurrent glioma infiltrating from adjacent structures, namely, the temporal lobe or pineal region, and 1 case with remote recurrence of parietal glioblastoma. These cases all died due to brainstem lesions, stressing the importance of developing an effective treatment strategy against brainstem disease. This study also recruited recurrent cases of DIPG, including 4 adult cases and 6 pediatric cases. Treatment at recurrence was quite difficult in pediatric DIPG cases, as 2 cases shown in Supplementary Figures 2 and 3. Over the last 30 years, multiple attempts have been made to improve the survival of brainstem glioma patients.^3,4^ One such strategy is local chemotherapy. However, the distribution of the drug to the lesion is restricted in local chemotherapy. CED offers an improved volume of distribution (*V*_d_) of therapeutic agents within the target site,^6^ and its potential has been demonstrated using targeted toxins,^13,14^ chemotherapeutic agents,^15,16^ and antisense oligonucleotides^17^ in preclinical and clinical settings. The most advanced trial to date was the “PRECISE trial,” which infused a fusion toxin that conjugates recombinant human IL-13 with mutant *Pseudomonas* exotoxin against recurrent glioblastoma.^18^ This study, however, failed to show statistically significant survival benefits over carmustine wafer implantation. One explanation for this failure is the insufficiency of drug distribution. It was estimated that coverage of the relevant target volumes was quite low at about 20%.^19^ Another explanation is the insufficient efficacy of the drug itself.^20^ With regard to local chemotherapy, it has been demonstrated that biodegradable drug delivery carriers left in the cavity after tumor resection enable the continuous release of chemotherapeutic agents and show efficacy, even though the penetration of carmustine is thought to be restricted to a small area.^21,22^ In this study, we applied this technique for the infusion of ACNU. Although nitrosourea compounds including ACNU are known to be effective, the use of nitrosourea compounds is limited by their toxicity.^23,24^ When delivered intravenously, the dose-limiting toxicity is myelosuppression. Therefore, local delivery is promising not only because it increases local concentration, but also because it decreases systemic exposure. ACNU is the most water-soluble of the nitrosoureas, making it most suitable for distribution by CED.^25^ Moreover, its efficacy is defined by the concentration rather than the exposure time.^26^ Another advantage of ACNU is that intrathecal delivery has already demonstrated that this agent is tolerable.^27,28^ Upon local delivery, ACNU is robustly distributed, similar to that achieved by Evans-blue.^9^ In addition, ACNU transiently opens the BBB.^29^

In this study, we also attempted to monitor drug distribution by mixing Gd-DOTA with the infused agent.^30^ As retention of Gd after the local infusion was insufficient, exact monitoring was not possible. However, it ensured the quality of CED by visualizing the flow of infusate within several hours of infusion.

The main objective of this study was to evaluate the toxicity of CED of ACNU combined with systemic TMZ. All 6 cases of recurrent malignant glioma and 9 cases of DIPG were already on TMZ at the time of recurrence. Many patients reported worsening of existing symptoms during infusion. However, most recovered within a week post-infusion to the pre-CED level. In the first 3 cases treated with 0.75 mg/mL concentration (5.25 mg dose), 2 developed adverse events that exceeded the transient period. A 31-year-old female experienced worsening of motor weakness and dysarthria but recovered within 2 months. Another case was a 40-year-old male with uncontrollable tumor growth, which made the determination of treatment-related symptoms and tumor growth-related symptoms difficult. Due to these events, we added 4 more cases to this group and found no additional long-lasting treatment-related adverse events. Although the protocol defined that the final dose given should be that at which 3 of 6 cases developed severe adverse events, and we only had 2 of 6 cases with such events, we decided to stop at this level. Rather than proceeding to a higher dose until we had more severe adverse events and then making the dose 1 level lower the maximum tolerable dose, the study stopped here defining the 0.75 mg/mL concentration (5.25 mg dose) as the maximum tolerable dose.

Antitumor efficacy was likely associated with dose level. At 0.25 mg/mL (1.75 mg dose), treatment failed to control tumor growth. At 0.5 mg/mL (3.5 mg dose), which was used in a previous case report demonstrating the disappearance of brainstem recurrent glioblastoma in a 14-year-old male,^31^ SD was the best response in this series. However, multiple objective responses were found at 0.75 mg/mL (5.25 mg dose), including both adult and pediatric DIPG cases. Even a rapidly enlarging tumor with deteriorating symptoms disappeared in a 32-year-old adult case, resulting in 33 months overall survival. Overall, the data generated in this study encourage us to further develop this strategy for the CED of ACNU.

However, this study has several limitations. First, this study included patients with recurrent gliomas that originated from non-brainstem sites (eg, cerebellum). These tumors likely differ from the brainstem and supratentorial gliomas, increasing the heterogeneity of this study population.^32^ Second, histology was not confirmed at recurrence which may dramatically impact the outcomes. Third, as this study included DIPG and malignant glioma diagnoses, there was a wide range in age, KPS, tumor locations, focality, prior treatments, and time from the original diagnosis.

Recently, Souweidane et al.^33^ reported the result of their phase 1 trial infusing radioimmunotherapy agent targeting the glioma-associated B7-H3 antigen against pediatric DIPG and demonstrated the safety and feasibility of this strategy. Our study included 6 pediatric DIPG cases (cases 8, 9, 10, 14, 15, 16), resulting in the median OS of 10.5 months, which is in the range of the median OS of this population reported elsewhere.^34^ As the sample size is too small to draw any conclusion about the efficacy against pediatric DIPG, subsequent study is planned to assess the efficacy in this populations. To date, alkylating agents including nitrosoureas are the only agents whose efficacy against gliomas is proven in clinical settings. This therapeutic strategy warrants further development for gliomas affecting the brainstem.

## Funding

This research was supported in part by the Japan Agency for Medical Research and Development (AMED) under grant number JP19lk0201070.

## 

*Conflict of interest statement*. None declared.

## Authorship Statement.

Study design: R.S. and T.T. Data collection: R.S., M.K., Y.S., K.N., and T.M. Data analysis: R.S., S.M., E.W.S., and R.T. Data interpretation: R.S., M.K., Y.Y., T.K., E.W.S., R.T., and T.T. All authors contributed to the writing and editing of the manuscript and agreed with the final version of the report.

Prior presentation presented in part at the Society for Neurooncology 2017 Annual Meeting, San Francisco, CA, USA.

## Supplementary Material

vdaa033_suppl_Supplementary_Figure_1

vdaa033_suppl_Supplementary_Figure_2

vdaa033_suppl_Supplementary_Figure_3

vdaa033_suppl_Supplementary_Material
