# Supplementary figures and images for: Phase I trial of convection-enhanced delivery of nimustine hydrochloride (ACNU) for brainstem recurrent glioma
Source: Neurooncol Adv. 2020 Mar 26;2(1):vdaa033. doi: 10.1093/noajnl/vdaa033 (PMC7212853; doi:10.1093/noajnl/vdaa033)

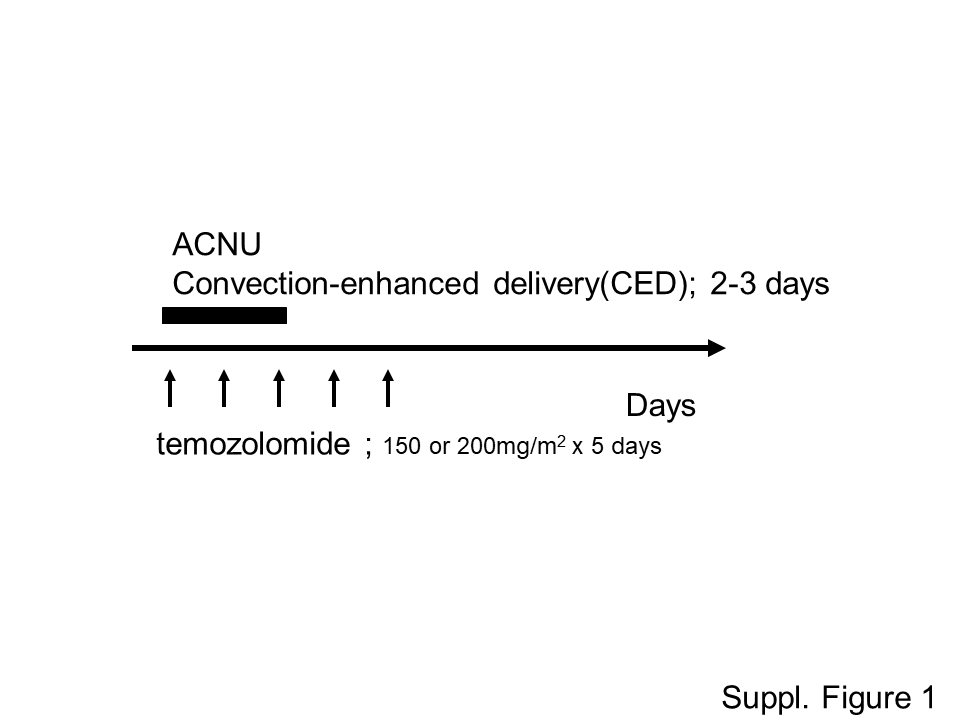

Supplement: vdaa033_suppl_Supplementary_Figure_1 [file vdaa033_suppl_supplementary_figure_1.png]

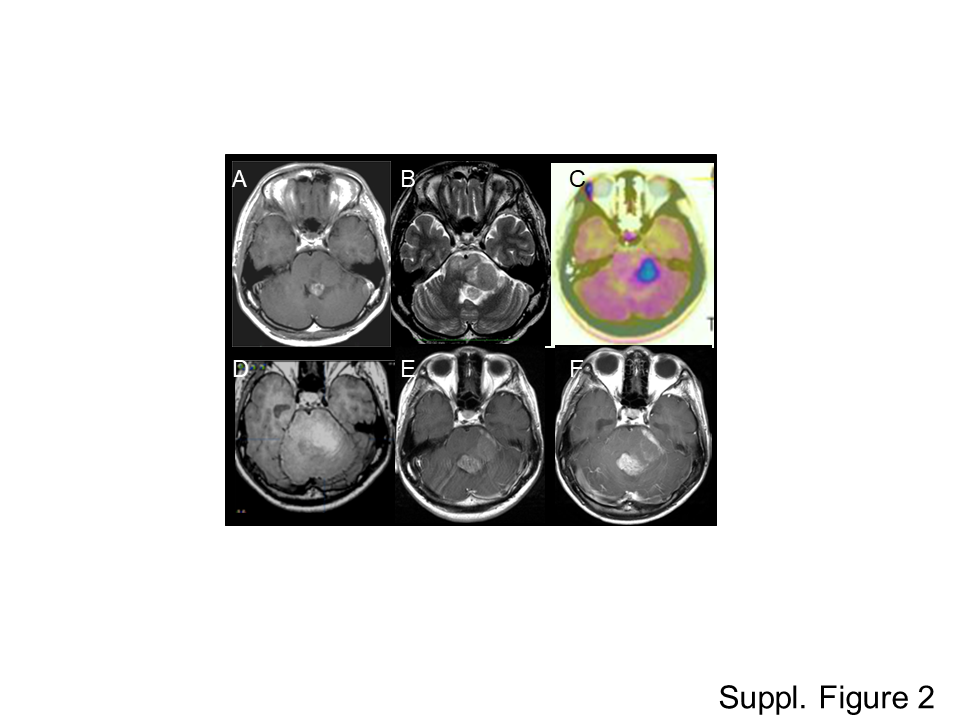

Supplement: vdaa033_suppl_Supplementary_Figure_2 [file vdaa033_suppl_supplementary_figure_2.png]

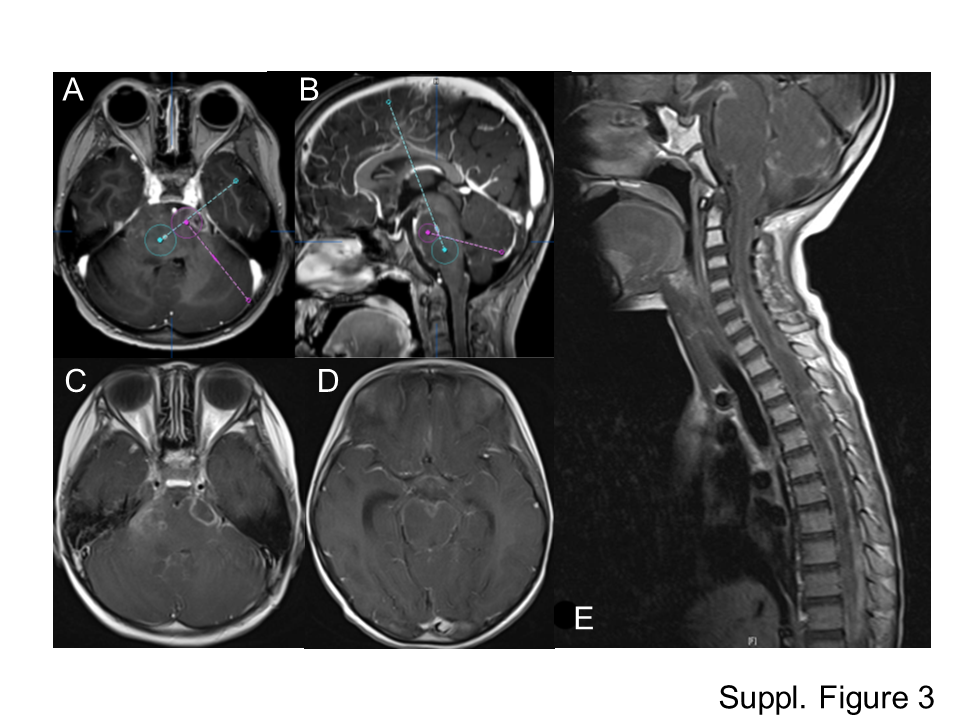

Supplement: vdaa033_suppl_Supplementary_Figure_3 [file vdaa033_suppl_supplementary_figure_3.png]
