# Supplementary material for: Phase I trial of convection-enhanced delivery of nimustine hydrochloride (ACNU) for brainstem recurrent glioma
Source: Neurooncol Adv. 2020 Mar 26;2(1):vdaa033. doi: 10.1093/noajnl/vdaa033 (PMC7212853; doi:10.1093/noajnl/vdaa033)
Supplement: vdaa033_suppl_Supplementary_Material [file vdaa033_suppl_supplementary_material.docx]

Supplementary Fig. 1 Schedule of treatment. Patients underwent stereotactic implementation of the catheter on day 1. After initial MRI examination, the catheter was connected to a microinfusion pump and each patient received continuous infusion of 7 mL ACNU at a speed of 1-5 μl/min. In addition to CED of ACNU, patients received systemic temozolomide following the protocol for recurrent tumors, namely 150 or 200 mg/m^2^/day for 5 days starting from day 1 of CED.

Supplementary Fig. 2 A case of 19-year-old male treated against DIPG for 22 months prior to starting CED of ACNU. He was first treated with local 54 Gy irradiation plus concomitant TMZ. However, recurrence was diagnosed 7 months before CED. Bevacizumab was used after recurrence in combination with TMZ. After the second relapse, he was referred to us for treatment (A,B). Positron emission tomography (PET) revealed high uptake of methionine at a slightly enhancing mass located at the left cerebellar peduncle (C). Despite the apparent enhancing mass protruding into the fourth ventricle, we decided to target the slight enhancing mass located at the left cerebellar peduncle. CED of ACNU was successfully delivered to the left cerebellar peduncle (D), but the patient died 2 months after this treatment due to enlargement of the enhancing mass protruding into the fourth ventricle (E: 1 week after CED; F: 1 month after CED). Autopsy revealed vital tumor both at the enhancing and drug-distributed area. A, B, E, and F: T1-weighted images with contrast enhancement; C: methionine PET; D: T1 weighted image detecting Gd-DOTA signal co-infused with ACNU.

Supplementary Fig. 3 A case of a 6-year-old. female treated for DIPG for 9 months before starting CED of ACNU. She underwent CED of ACNU at the first recurrence, however, she developed urinary retention just 2 weeks after treatment. Catheter tracts for CED (A,B). Brain and spinal MRI revealed massive intracranial and spinal dissemination. (C,D) T1-weighted brain images with contrast enhancement. (E) T1-weighted spinal image with contrast enhancement.
